# Community Vertical Composition of the Laguna Negra Hypersaline Microbial Mat, Puna Region (Argentinean Andes)

**DOI:** 10.3390/biology11060831

**Published:** 2022-05-28

**Authors:** Flavia Jaquelina Boidi, Estela Cecilia Mlewski, Guillermo César Fernández, María Regina Flores, Emmanuelle Gérard, María Eugenia Farías, Fernando Javier Gomez

**Affiliations:** 1 Laboratorio de Biología Molecular, Hospital SAMCo Rafaela, 737 Lisandro de la Torre, Rafaela 2300, Argentina; 2 Instituto Multidisciplinario de Biología Vegetal (IMBIV), Facultad de Ciencias Exactas, Físicas y Naturales, Universidad Nacional de Córdoba, CONICET, 1666 Vélez Sarsfield Av., Cordoba 5000, Argentina; ecmlewski@imbiv.unc.edu.ar; 3 Centro de Investigaciones en Ciencias de la Tierra (CICTERRA), Facultad de Ciencias Exactas, Físicas y Naturales, Universidad Nacional de Córdoba, CONICET, 1699 Vélez Sarsfield Av., Cordoba 5000, Argentina; guillefernandez.unc@gmail.com; 4 Department of Research and Development, PROMICOL B.V., 12 De Asselen Kuil, 6161 Geleen, The Netherlands; acm_regy@hotmail.com; 5 Institut de Physique du Globe de Paris, Université de Paris Cité, CNRS, F-75005 Paris, France; emgerard@ipgp.fr; 6 Planta Piloto de Procesos Industriales y Microbiológicos (PROIMI), Centro Científico Tecnológico, CONICET, Belgrano Av. & Pasaje Caseros, San Miguel de Tucuman 4000, Argentina; mefarias2009@gmail.com

**Keywords:** microbial diversity, pigments, extreme environment, UV radiation, high-altitude, hypersaline lake, Andes, Puna region

## Abstract

**Simple Summary:**

The Laguna Negra is a high-altitude hypersaline lake located in the Puna region in Argentina, hosting microbial mats and modern stromatolites, microbially formed rocks found throughout the geological record. Some recent studies have shown the relevance of the lakes in this region for (i) the discovery of new extremophiles, particularly those tolerant to high UV and, furthermore, (ii) the study of biosignatures in stromatolites. In this work, a detailed survey, layer by layer, of the microbial diversity of the most widespread microbial mat in the Laguna Negra was reported, to unravel the spatial arrangement of the microbial community. The results reveal the vertical distribution of the main prokaryotic microbial taxa, and underline abundant Deinococcus-Thermus at the top of the microbial mat with high amounts of deinoxanthin, which may help the total community to cope with high UV radiation. This study allows a better understanding of the community strategies to thrive under environmental stressors in the Laguna Negra and its counterparts in the Altiplano-Puna region.

**Abstract:**

The Altiplano-Puna region is a high-altitude plateau in South America characterized by extreme conditions, including the highest UV incidence on Earth. The Laguna Negra is a hypersaline lake located in the Catamarca Province, northwestern Argentina, where stromatolites and other microbialites are found, and where life is mostly restricted to microbial mats. In this study, a particular microbial mat that covers the shore of the lake was explored, to unravel its layer-by-layer vertical structure in response to the environmental stressors therein. Microbial community composition was assessed by high-throughput 16S rRNA gene sequencing and pigment content analyses, complemented with microscopy tools to characterize its spatial arrangement within the mat. The top layer of the mat has a remarkable UV-tolerance feature, characterized by the presence of Deinococcus-Thermus and deinoxanthin, which might reflect a shielding strategy to cope with high UV radiation. Chloroflexi and Deltaproteobacteria were abundant in the second and third underlying layers, respectively. The bottom layer harbors copious Halanaerobiaeota. Subspherical aggregates composed of calcite, extracellular polymeric substances, abundant diatoms, and other microorganisms were observed all along the mat as the main structural component. This detailed study provides insights into the strategies of microbial communities to thrive under high UV radiation and hypersalinity in high-altitude lakes in the Altiplano-Puna region.

## 1. Introduction

Microbial mats are layered microbial communities embedded in extracellular polymeric substances (EPS) developed at the sediment–water interface, and their vertical stratification responds to light availability and chemical gradients, mostly oxygen and sulfide [1,2,3]. Microbial mats develop in a variety of habitats; however, they are particularly common where predation and competition with other organisms are limited [4]. Therefore, such settings for microbial mats growth are provided by environments considered as extreme in terms of salinity, UV radiation influx, pH, and temperature [5,6].

The Altiplano-Puna plateau in the Central Andes is the second highest plateau in elevation on Earth after Tibet and it encompasses northwestern Argentina, southwestern Bolivia, northeastern Chile, and southeastern Peru [7,8]. The region is characterized by extreme environmental conditions, including high solar and UV radiation, aridity, great seasonal variations in precipitation, strong winds, and wide daily temperature range [9,10,11,12]. Due to (i) an ozone column naturally thinner over the tropics, (ii) UV increment with elevation, (iii) clear skies, and (iv) low aerosols [13], the solar and UV irradiance in the Altiplano-Puna are the highest in the world. UV Index values can exceed 20 in the region [14], and record spikes have been registered in the Atacama Desert, Chile [15], Laguna Lejía, Chile [16], Cuzco, Peru [14], and Licancabur, Bolivia [17]. The UV flux in the Altiplano-Puna is 165% of that measured at sea level with a maximum averaged UV-B up to 4 W/m^2^ and short UV wavelengths incidence (260–270 nm) peaks at 14.6 mW/m^2^ on the ground [17,18].

The Laguna Negra (LN) in the Catamarca Province, Argentina, is one of the High-Altitude Andean Lakes (HAAL); saline-hypersaline shallow lakes located at altitudes from 3000–6000 m.a.s.l. in the Altiplano-Puna region. Many HAALs harbor recently reported outstanding microbial mats and microbialites systems, including stromatolites [19,20,21,22,23,24,25,26]. Interestingly, studies of these ecosystems have led to the discovery of novel poly-extremophiles, tolerant to high UV radiation and hypersalinity, particularly bacterial and archaeal UV-tolerant strains [27,28,29,30,31,32,33,34,35,36,37]. For instance, the UV-tolerant and metal- and metalloids-resistant bacterium *Exiguobacterium chiriqhucha* str. N139 is a new poly-extremophile retrieved from the LN [38].

Previous studies described the diversity of different microbial mats from the LN; however, the communities were studied at a bulk scale, without discriminating the diverse internal layers [39,40]. The present study focuses on each pigmented layer of the most widespread stratified microbial mat, in order to have a more detailed understanding of the vertical distribution of key phylogenetic and functional groups. First, the bacterial and the so far unknown archaeal composition of each individual horizon of the mat were revealed by high-throughput sequencing of V4 region of the 16S rRNA gene. Secondly, pigments were identified to complement the diversity data and provide information about light-related microbial metabolisms and capabilities. Moreover, confocal laser scanning microscopy (CLSM) and scanning electron microscopy (SEM) were used to unravel the microbes–minerals arrangement in the mat. Lastly, energy dispersive X-ray spectroscopy (EDS) and X-ray diffraction (XRD) analyses were performed to characterize the mineralogy and chemical composition of precipitates. The obtained results considerably expand our knowledge of the microbial diversity present in this extreme environment, providing insights into strategies of microbial communities to thrive in hypersaline high-altitude lakes in the Altiplano-Puna region.

## 2. Materials and Methods

### 2.1. Study Site 

**Figure 1:**
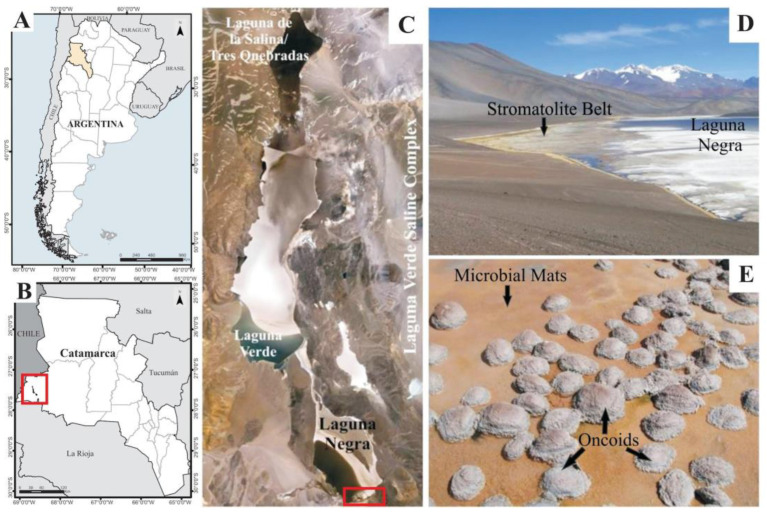
Geographical location of the studied area. (**A**) Argentina map showing the Catamarca Province in yellow; (**B**) Catamarca Province map showing the location of the Laguna Verde Saline Complex (red square); (**C**) Laguna Negra satellite image showing the study area location (red square); (**D**) Overview of the “Stromatolite Belt” in the southeastern shore of the Laguna Negra, where microbialites and microbial mats occur; (**E**) A detailed image of microbial mats surrounding oncoids in the “Stromatolite Belt”.

The LN is located at the southeast edge of the Laguna Verde Complex (LVC, GPS 27°38′49″ S, 68°32′43″ W), in the Puna region of Catamarca Province, Argentina, and placed at 4500 m.a.s.l. (Figure 1A–C). The geography and climate are typical of the high-altitude Puna plateau in the Andes, dominated by volcanic and volcaniclastic rocks, strong winds, a wide daily and seasonal range of temperatures, high solar radiation, negative hydrological balance, and abundant evaporite precipitation [19]. The LN is a shallow hypersaline lake rich in CaCl_2_ ([Ca^2+^] = 0.4 M; [Cl^−^] = 5.6 M), with a salinity of ~320 ppt [19]. The lake hydrogeological setting is complex, receiving both surface and groundwater springs inputs, resulting in a mixing zone oversaturated with different calcium carbonate mineral phases, mainly calcite and aragonite. This zone along the southeastern shore of the lake was named the “Stromatolite Belt”, and consists of oncoids, stromatolites, laminar crusts, and associated microbial mats (Figure 1D,E) [19,39].

### 2.2. Sample Collection

**Figure 2:**
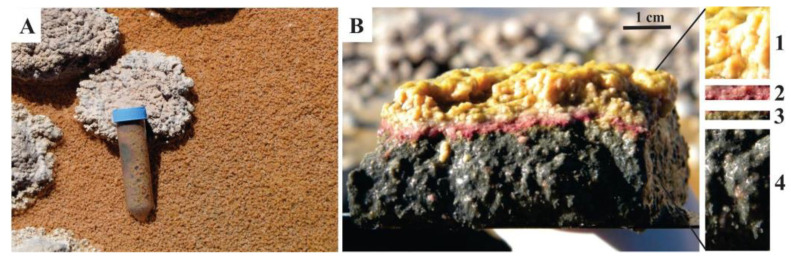
Studied microbial mat. (**A**) Overview; (**B**) Vertical section of one portion of the microbial mat showing the four dissected layers: pink-orange Layer 1, purple Layer 2, green Layer 3, and black Layer 4.

Within the Stromatolite Belt, a predominant subaqueous layered microbial mat that covers the sediment is observed, mostly associated with oncoids (Figure 1E). Its macroscopic aspect shows a stratified internal structure. Samples analyzed in this study were collected in 2013 (Southern Hemisphere, early autumn, water temperature 12 °C), and were dissected based on the different colors observed in a depth profile, referred to as Layer 1, 2, 3, and 4 (Figure 2). Layer 1, at the top, was pink-orange, occasionally yellowish-golden colored, with a granular appearance, and 3–6 mm thick. Layer 2 was thinner than the previous one (1–2 mm in thickness), purple, and showed a homogeneous texture. Layer 3 was green, very cohesive with Layer 2, and measured 1–2 mm in thickness. Layer 4 was thicker (up to 1 dm), black, and homogeneous. Only the upper 1 cm of the Layer 4 was sampled for the present work. 

Five portions of the microbial mat were taken from within an area of ~0.50 m^2^ from one sampling site to accommodate potential patchiness in the strata (same as in Harris et al. [41]). Each portion was sectioned very carefully with sterile scalpels at the boundaries of its colored layers and the interfaces between them were avoided to prevent any mixture, a procedure that was followed in the past by similar studies in microbial mats (e.g., [41,42]). The five subsamples from each layer retrieved from the portions were pooled for consecutive analyses, resulting in one final sample per layer. Samples for DNA extraction were separated and stored at −20 °C until processing within a week. Samples for high-performance liquid chromatography (HPLC) were separated, lyophilized immediately, and stored at −20 °C. Samples for methanol pigment extraction were kept in complete darkness, free of oxygen and at −20 °C until processing. Samples for confocal laser scanning microscopy (CLSM) were immediately fixed in RNAlaterTM (Thermo Fisher Scientific, Waltham, MA, USA) and stored at −20 °C until use. Samples for scanning electron microscopy (SEM) were kept at 4 °C in the field and then fixed in the laboratory with glutaraldehyde 2% (G5882, Sigma-Aldrich, St. Louis, MO, USA).

### 2.3. DNA Extraction and 454 Pyrosequencing

Total genomic DNA was extracted from each sectioned layer using the Power Biofilm DNA Extraction Kit (MoBio, Carlsbad, CA, USA). In all cases, 0.1 g of homogenized material was processed according to manufacturer’s instructions. The V4 hyper-variable region of the 16S rRNA gene was amplified from total DNA using the primers RK-TAG 515F and 806R [43], which allow assessing the diversity of both Bacteria and Archaea. The primers contained the Roche 454 sequencing A and B adaptors and a 10 nucleotide “multiple identifier” (MID).

PCR amplification was conducted on a FastStart Fidelity PCR system (Roche Applied Science, Mannheim, Germany) following the manufacturer’s protocol. Five independent PCRs were carried out to reduce bias. Two negative controls with no template were also performed. The PCR mixture contained 2.5 µL FastStart High Fidelity 10X Reaction Buffer, 20 ng of template DNA, 0.4 µM of each primer, 1.25 U FastStart High Fidelity Enzyme Blend (Roche Applied Science, Mannheim, Germany), and 0.2 mM dNTPs. The PCR conditions were 95 °C for 5 min, followed by 30 cycles of 95 °C for 45 s, 57 °C for 45 s, and 72 °C for 60 s, and a final elongation step at 72 °C for 4 min. The five reaction products were pooled and purified using AMPure beads XP (Beckman Coulter, Indianapolis, IN, USA). Quantification of the purified PCR products was performed using Quant-IT Pico Green dsDNA Kit (Invitrogen Molecular Probes Inc., Eugene, OR, USA). Amplicons were sequenced on the Roche 454 Genome Sequencer FLX pyrosequencing platform at the INDEAR facilities (Rosario, Argentina), following the amplicon sequencing protocol provided by the manufacturer.

### 2.4. Data Analysis 

Analysis of the 16S rRNA gene pyrotags was initially performed using the Quantitative Insights Into Microbial Ecology (QIIME) software package v.1.7.0. [44]. Reads were split into the corresponding sample using the split_libraries.py script implemented in QIIME. Reads that had a mean quality score < 25, a maximum homopolymer run > 6, a number of primer mismatches > 10, and a read length < 200 bp or >1000 bp were discarded. Primers were removed using the CUTADAPT software v.2.4 [45]. Subsequent data were imported to QIIME 2 software [46]. Sequences were denoised and filtered and chimeras were removed using the q2-DADA2 v.2019.7.0 software [47]. In order to obtain high-quality data, single-end reads were truncated at position 250. The resulting Amplicon Sequence Variants (ASVs) were used to calculate alpha diversity parameters by the q2-diversity plug-in. Rarefaction curves were obtained using the alpha rarefaction plug-in implemented in QIIME 2. Data were rarefied at 3250 sequences per sample. The alpha diversity indices calculated in this study include Observed ASVs, Chao1, Dominance, Pielou_e, Shannon, and Simpson. Taxonomic composition was assigned using a pre-trained Naive Bayes classifier on the SILVA database (release 132–99% OTUs, full-length sequences) with the q2-feature-classifier plug-in. Venn diagram and families heatmap were generated in R v.3.6.3 [48]. Most abundant ASVs taxonomic affiliations were further evaluated by BLAST search against the NCBI nucleotide collection (nt) database. The sequences were deposited as FASTQ in the NCBI Sequence Read Archive (SRA) under the accession number PRJNA564857.

### 2.5. Pigment Identification 

Preliminary pigment analysis was done following the conventional methanol protocol [49]. Sample aliquots of 5 g were placed in centrifuge tubes (50 mL) with 10 mL of absolute methanol (n° cat. 711, Cicarelli, San Lorenzo, Santa Fe, Argentina). Tubes were placed in a thermostatic bath for 1 h at 45 °C and then stored at 4 °C overnight. After the extraction period, the samples were centrifuged (15 min at 10,000 rpm and 4 °C) and then 3 mL of the supernatant were taken and scanned (250–800 nm) in a UV-1800 UV/VIS Rayleigh spectrophotometer using a 1-cm path-length quartz cuvettes.

Pigment content was also analyzed by HPLC [50]: 150 mg of lyophilized material was incubated overnight at darkness and 4 °C, with 1.5 mL of absolute methanol. Then, samples were centrifuged at 8000× *g*, 4 °C for 10 min, and supernatants were filtered with 0.2 μm pore-diameter syringe filters before HPLC processing. The HPLC system consisted of two pumps (model 510, Waters, Mildford, MA, USA), a syringe loading injector (Rheodyne 7125, Idex Health and Science LLC, Rohnert Park, CA, USA) fitted with a 200 µL loop (Rheodyne 7025, Idex Health and Science LLC, Rohnert Park, CA, USA), and an on-line detection by diode array-based spectroscopy between 250 and 800 nm (996, Waters, Mildford, MA, USA). It was coupled with a computer equipped with the Empower 2007 Chromatography Manager software (Waters, Mildford, MA, USA), allowing for the detection of pigment spectra. The column used was 100 × 4.6 mm Kinetex C-18 (3 µm silica particle size) protected by an Ultra In-Line Krudkatcher filter (Phenomenex, Torrance, CA, USA). Pigments were identified by comparing the peak retention times and the corresponding absorption spectra against standards available in the laboratory or, when not available, against data in the LipidBank database [51]. Pigment abundance was quantified based on the peak areas in the chromatograms measured at an absorption wavelength of 435 nm. Peak delimitation and area integration were carried out automatically by the instrument’s software. Since most of the detected pigments lacked standards, their peak areas were normalized to the highest area of known peaks. Because all samples were collected and analyzed in the same way, the relative pigment abundances can be compared directly in all samples. In addition, Chlorophyll *a*, Beta-carotene, Lycopene, Diatoxanthin, Lutein, Canthaxanthin, and Astaxanthin were identified and quantified using standards from DHI, Denmark.

### 2.6. Scanning Electron Microscopy and Mineralogy

Glutaraldehyde-fixed samples were dehydrated in a gradual series of ethanol (n° cat. 748, Cicarrelli, San Lorenzo, Sta Fe, Argentina) and water baths at increasing ethanol concentrations (i.e., 10, 30, 50, 70, and 100%), air dried, and coated with gold. SEM analyses were performed with a field emission Zeiss Sigma scanning electron microscope (Carl Zeiss NTS GmbH, Oberkochen, Germany) at the X-ray Analysis Laboratory (LAMARX Universidad Nacional de Córdoba, Argentina). In addition, a subsample was dried at the CO_2_ critical point, carbon-coated and observed at the Service Commun de Microscopie Electronique à Balayage (IMPMC, Sorbonne University, Paris, France) using a Zeiss Supra 55VP (Carl Zeiss NTS GmbH, Oberkochen, Germany) scanning electron microscope equipped with an energy dispersive X-ray spectrometer for energy dispersive X-ray spectroscopy (EDS) (X flash Quad detector, Bruker, Billerica, MA, USA).

In order to find out whether the mineral composition changes throughout the microbial mat, layers were dried and finely ground (<20 µm) to determine their mineralogical composition by X-ray diffraction (XRD) using a Philips X’PERT PRO diffractometer and a Cu lamp (kα = 1.5406 Å) operated at 40 mÅ and 40 kV housed within the Departamento de Cristaloquímica, Facultad de Ciencias Químicas, Universidad Nacional de Córdoba.

### 2.7. Imaging by Confocal Laser Scanning Microscopy

Images of fixed-samples stained with Syto^®^9 (Thermo Fisher, Waltham, MA, USA), a green fluorescent nucleic acid stain, were obtained using a FluoViewTM FV1000 confocal laser scanning microscope with a spectral resolution of 2 nm and a spatial resolution of 0.2 μm (Olympus, Tokyo, Japan) at the Institut de Physique du Globe de Paris (IPGP). The FluoViewTM 10 FV1000 was equipped with a 405 nm laser diode, and multi-line argon (458 nm, 488 nm, and 515 nm), helium-neon-green (543 nm), and helium-neon-red (633 nm) lasers. Fluorescence images were obtained with concomitant excitation at wavelengths of 405 nm, 488 nm, and 633 nm by collecting the emitted fluorescence between 425–475 nm, 500–600 nm, and 655–755 nm, respectively. Three-dimensional images were acquired, visualized, and processed using the F10-ASW FLUOVIEW and ImageJ 1.8.0_172 software (National Institutes of Health, Bethesda, MD, USA) [52].

## 3. Results

### 3.1. Prokaryotic Microbial Diversity

**Figure 3:**
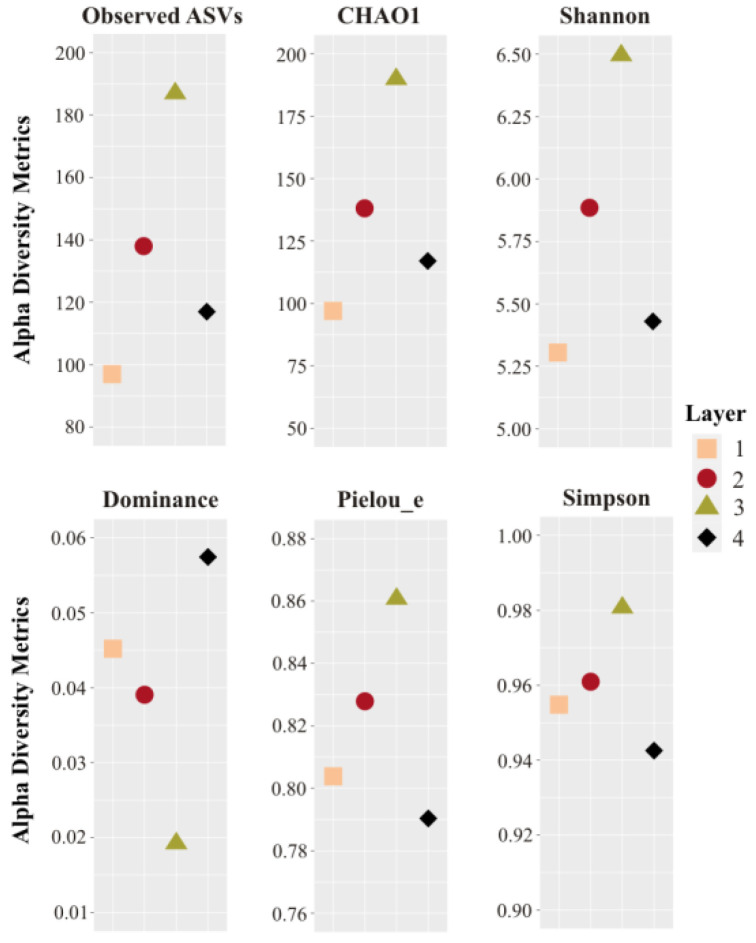
Alpha diversity metrics of the four layers studied. Sampling effort was normalized at 3250 reads per sample. ASVs: amplicon sequence variants.

Overall, after quality filtering, a total of 14,984 sequences were obtained from all sampled layers. In total, 257 reads corresponding to chloroplasts and mitochondria were discarded for the diversity analysis. Rarefaction curves showed that all the samples reached a plateau, i.e., sequencing depth saturation, suggesting that microbial diversity was sufficiently covered by the sequencing effort (Supplementary Figure S1). Observed richness and diversity estimates based on ASVs were compared between the layers (Figure 3, and Supplementary Tables S1–S6). The highest CHAO1, Pielou_e, Observed ASVs, and Shannon values were found in the middle Layers 2 and 3, particularly Layer 3, along with the lowest Dominance index, revealing rich and even communities. The lowest CHAO1, Observed ASVs, and Shannon values were found in Layers 1 and 4, especially in Layer 1. The highest Dominance was found in Layer 4, followed by Layer 1 (Figure 3).

**Figure 4:**
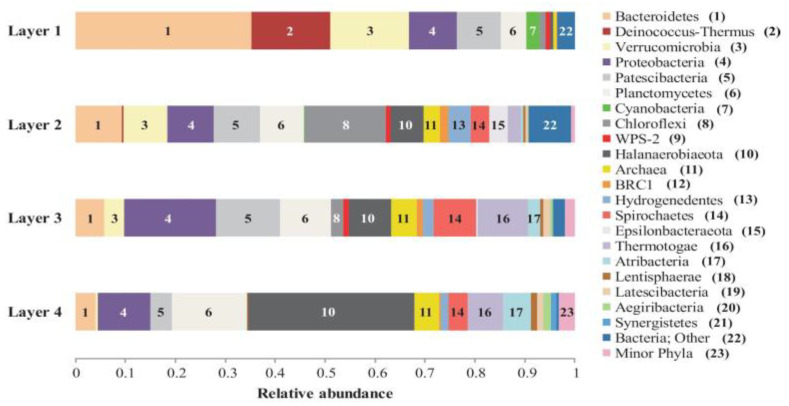
Relative abundance of bacterial phyla and Archaea in the Laguna Negra microbial mat layers. Assignations are derived from sequences analysis of the V4 hypervariable region of 16S rRNA gene. “Minor phyla” indicates less than 1% of abundance and included Acetothermia, Acidobacteria, Chlamydiae, Cloacimonetes, Firmicutes, Fusobacteria, Marinimicrobia (SAR406 clade), Omnitrophicaeota, and WS1. “Bacteria; Other” indicates unclassified.

**Figure 5:**
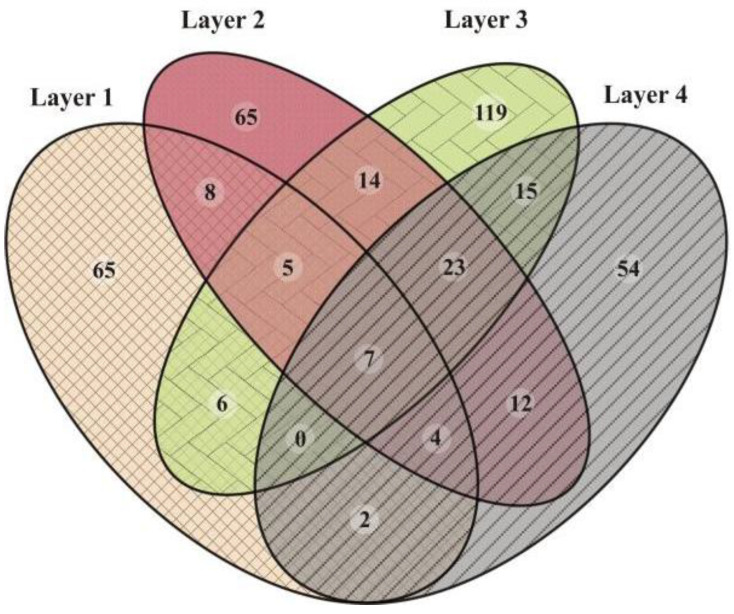
Venn diagram showing the number of unique or shared ASVs between the layers of the microbial mat.

**Figure 6:**
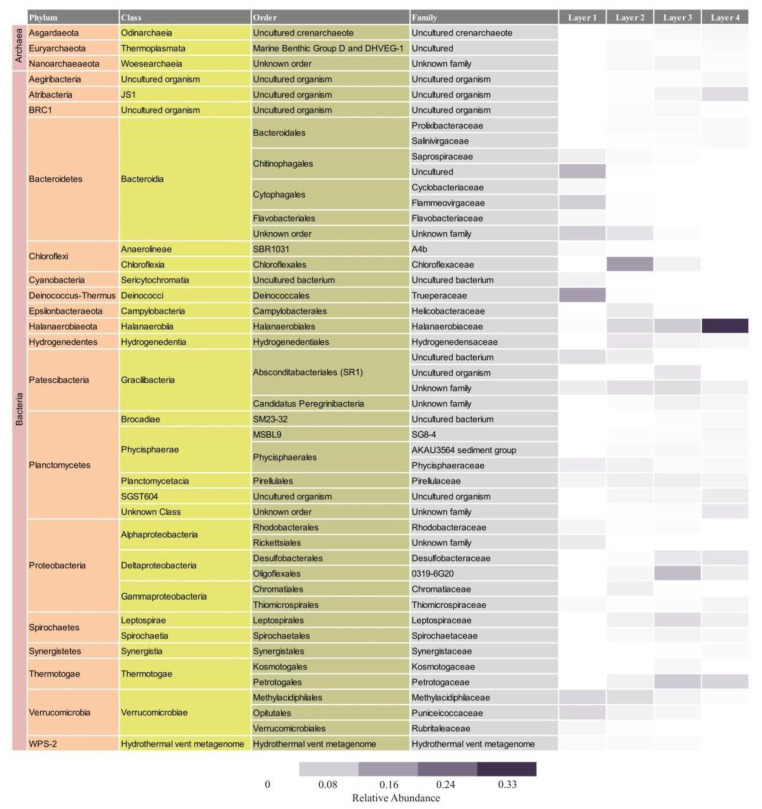
Relative abundance and taxonomic assignment of the main families found in each layer. The figure shows families with relative abundances higher than 1% of overall dataset.

The microbial composition of Layer 1 was the most different compared to the other layers, with Bacteroidetes (35%), Deinococcus-Thermus (16%), Verrucomicrobia (16%), Proteobacteria (10%), and Patescibacteria (9%) as the most abundant phyla (Figure 4), and the highest proportion of unique ASVs (Figure 5). Bacteroidetes were entirely represented by the Bacteroidia class (Figure 6), and 12% of the total reads from Layer 1 belonged to one ASV with no further classification other than the Chitinophagales order (Supplementary Table S7). Within the Cytophagales order, Flammeovirgaceae family, *Flexithrix* sp. comprised 6% of the total reads of Layer 1 (Figure 6 and Supplementary Table S7). All sequences from Deinococcus-Thermus belonged to the Trueperaceae family (Figure 6), and the two most abundant ASVs corresponded to the *Truepera* sp. genus, 8% and 7% of the total reads each and their best BLAST hit were hyperhalophilic uncultured bacteria (Supplementary Table S7). Members of Verrucomicrobia included Methylacidiphilaceae and Puniceicoccaceae families, with one ASV from this last family (3% of Layer 1 total reads) assigned to *Lentimonas* sp., and BLAST related to a marine uncultured bacterium (Figure 6 and Supplementary Table S7). Cyanobacteria were present in Layer 1, with 3% of the total reads clustered in one ASV within the Sericytochromatia class (Figure 6 and Supplementary Table S7).

Layer 2 presented abundant Chloroflexi (16%), followed by Bacteroidetes (9%), Verrucomicrobia (9%), Proteobacteria (9%), Patescibacteria (9%), Planctomycetes (9%), and Halanaerobiaeota (7%) (Figure 4). Remarkably, Layer 2 presented the highest proportion of unclassified sequences (8% of the total reads) (Figure 4), and one prevalent ASV without taxonomic assignation (6% of the total reads) (Supplementary Table S7). Almost all Chloroflexi in Layer 2 affiliated to *Candidatus* Chlorothrix sp. (15% of the total reads) (Supplementary Table S7). There were two abundant ASVs in Layer 2 assigned to the Patescibacteria phylum, Absconditabacteriales (SR1) order, with 4% and 3% of the total reads each (Supplementary Table S7). Among the Epsilonbacteraeota phylum, there was an abundant ASV (2%) assigned to the Helicobacteraceae family. Other abundant families in Layer 2 included Hydrogenedensaceae (Hydrogenedentes phylum) and Chromatiaceae (Gammaproteobacteria) (Figure 6).

The main phyla in Layer 3 were Proteobacteria (18%), Patescibacteria (13%), Planctomycetes (10%), Thermotogae (10%), Halanaerobiaeota (9%), and Spirochaetes (9%) (Figure 4). Deltaproteobacteria were the main Proteobacteria class in this layer, with Desulfobacteraceae and 0319-6G20 (Oligoflexales) families as the most relevant (Figure 6). There were two abundant ASVs classified as 0319-6G20, with 3% and 7% of the total reads (Supplementary Table S7). Members of Patescibacteria reached taxonomic classification till order level, assigned as Absconditabacteriales (SR1) and Candidatus Peregrinibacteria (Figure 6). There were two distinct ASVs associated to genus SC103, Petrotogaceae family within the Thermotogae phylum, with 3% and 4% of the total reads, respectively (Supplementary Table S7). The Leptospiraceae family within the Spirochaetes phylum reached its highest abundance in Layer 3, with 3% of the total reads from this layer clustered in one ASV (Supplementary Table S7).

Layer 4 was rich in Halanaerobiaeota (33%), Planctomycetes (15%), and Proteobacteria (10%), followed by Thermotogae (7%), Atribacteria (6%), and Domain Archaea (5%) (Figure 4), and presented the lowest proportion of unique ASVs (Figure 5). All reads classified as Halanaerobiaeota belonged to the Halanaerobiaceae family (Figure 6), and three distinct ASVs were assigned to *Halanaerobium* sp. representing 19%, 9%, and 3% of the total reads from this layer. These three abundant ASV had their best BLAST matches with an uncultured *Halanaerobium* retrieved from hypersaline environments, and the *Halanaerobium praevalens* strain GSL isolated from Great Salt Lake sediment (Supplementary Table S7). Among Planctomycetes, there was an ASV classified as class SGST604, with 3% of relative abundance in this bottom layer, and another ASV without further classification than phylum, representing 2% of the total reads in Layer 4 (Supplementary Table S7). The Thermotogae phylum was represented in this layer by the Petrotogaceae family, with one ASV classified as the candidate genus SC103 (2% relative abundance of the total reads from this layer) (Figure 6 and Supplementary Table S7). Atribacteria consisted of the JSI class, with one abundant ASV comprising 4% of the total reads from this layer (Supplementary Table S7).

### 3.2. Pigments Analyses

**Figure 7:**
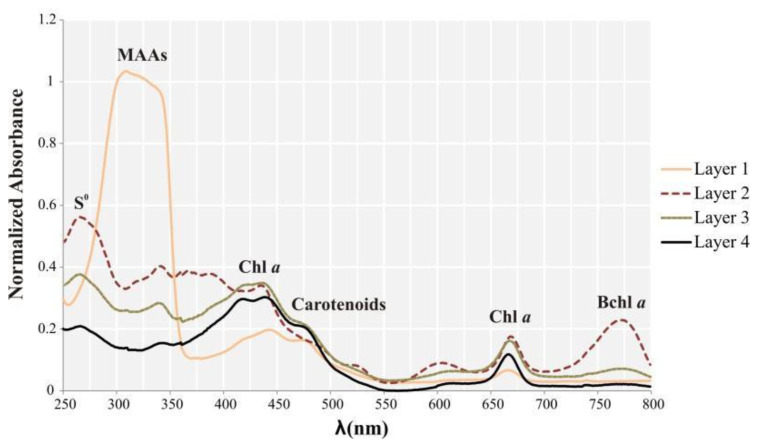
Total absorbance spectra of the pigments extracted with methanol from the four layers individually.

Pigments of Layer 1 showed a broad and intense absorbance peak in the UV region, between 280 and 350 nm. This may be indicative of diverse candidates, such as residual DNA, ubiquinone, and mycosporine-like amino acids (MAAs) (Figure 7). A peak centered approximately at 260 nm was detected in the layers below Layer 1, especially Layer 2. This absorbance peak corresponds to elemental sulfur, easily extracted with organic solvents [53], and may indicate sulfur storage by sulfur bacteria. Between 400 and 550 nm, all samples showed a high and broad peak, although in Layer 1, this peak was lower, and a shoulder was observed at 470 nm. Many pigments absorb in this wavelength range: chlorophylls (at 430 nm), cytochromes, and many carotenoids (around 470 nm), such as astaxanthin, cataxantin α, and β carotenes [54,55]. Chlorophyll *a* was found in all samples based on peaks at around 430 nm and 665 nm. Based on the height of the peaks, it was more abundant in Layers 2, 3, and 4. Layer 2 showed a high peak at 770 nm, which is interpreted as absorbance by bacteriochlorophyll *a*. Additional pigments, such as Bchl *c*, were likely present, but their absorbance peaks may overlap with those of more intensely absorbing pigments, and therefore, become hidden.

All pigments found by HPLC are summarized in Table 1. In Layer 1, at least eight photosynthetic pigments were detected. Among them, deinoxanthin, carotenoid, and cantaxanthin were exclusively found in Layer 1. Carotenoids give the layer its distinctive pink-orange color. Deinoxanthin is a carotenoid with a strong antioxidant activity retrieved from *Deinococcus radiodurans* [56]. Cantaxanthin is also a carotenoid present in cyanobacteria and other photosynthetic microorganisms such as diatoms, which were found in this layer. Additional pigments were detected in Layer 1, such as fucoxanthin (also present deeper in the mat), diadinoxanthin, a major carotenoid in diatoms also found in less quantity in Layer 2, diatoxanthin (present in all layers), zeaxanthin (also present in Layer 3), and chlorophyll *a*, essential for photosynthesis in diatoms and cyanobacteria (also found in Layer 3 and 4). In Layer 2, eight peaks related to pigments were observed. It was the only layer that showed (2S, 29S)-oscillol 2,29-difucoside (match 96%), a cyanobacterial pigment [57], and betacarotene. Bacteriochlorophylls *a*, *d*, and *c* were noteworthy in Layer 2, supporting pyrosequencing analyses which showed high proportions of anoxygenic phototrophs such as Chloroflexi and representatives of Gammaproteobacteria in this layer. Other pigments present in this layer in lesser amount were diadinoxanthin, diatoxanthin, and derivatives of bacteriochlorophyll *d*. In Layer 3, fucoxanthin, diatoxanthin, and bacteriochlorophyll *c* were identified. Bacteriochlorophyll *d*, zeaxanthin, chlorophyll *a*, and a derivative of bacteriochlorophyll *a*, possibly a degradation product, were detected in lower quantity. Derivatives of bacteriochlorophyll *d* and bacteriochlorophyll *a* were present in minor quantities. Layer 4 is the only one that contained pheophytin, a degradation product of chlorophyll. Chlorophyll *a*, a derivative of bacteriochlorophyll *a*, diatoxanthin, and fucoxanthin, and to a lesser extent bacteriochlorophylls *a* and *c*, and an unknown pigment were also found in Layer 4.

### 3.3. Scanning Electron Microscopy and XRD Mineralogy

**Figure 8:**
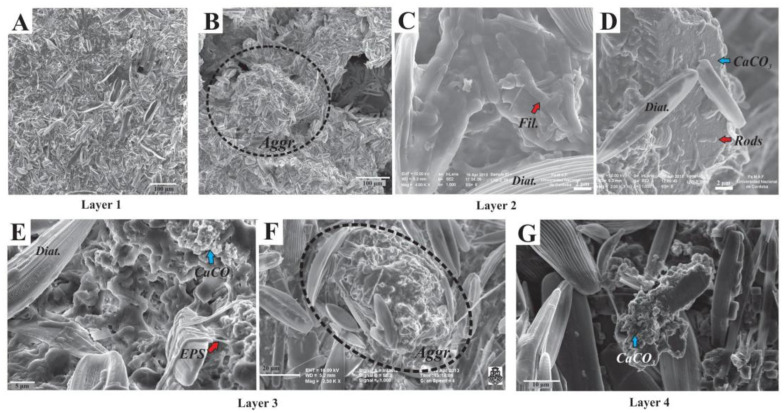
SEM images of the studied microbial mat layers. (**A**) Abundant pennate diatoms (image from Layer 1); (**B**) Irregular-subspherical diatom-microbe-mineral aggregates (image from Layer 2); (**C**,**D**) Filamentous and rod-shaped microbes within the EPS matrix together with mineral particles; (**E**) Diatoms and microbes immersed within the EPS matrix where clusters of irregular-globular mineral particles are also observed (blue arrow). Note the fibrillar textures of EPS (red arrow); (**F**) Subspherical aggregates where diatoms, EPS, and mineral remains are observed (picture from Layer 3); (**G**) Irregularly shaped mineral cluster where subhedral carbonate mineral particles are observed (image from Layer 4).

Diatoms were observed dispersed throughout the mat (Figure 8) as well as part of irregular-subspherical aggregates (20–300 µm diameter, Figure 8B,F), which were detected in all sampled layers. These subspherical aggregates were composed of (i) diatoms (mostly pennate), (ii) filamentous, coccoid, and rod-shaped microbes (Figure 8C,D), and (iii) calcium carbonate minerals within an EPS matrix (Figure 8D,E,G). Aggregates occasionally coalesced to form bigger aggregates or wavy-irregularly shaped horizontal lamina. Although diatoms were usually well preserved, fragmented and corroded frustules remains were locally observed within the EPS matrix, particularly in the lower layers. When observed in the EPS matrix, minerals were anhedral-subhedral (typically subspherical-globular carbonate particles, 80–700 nm) (Figure 8E–G), but subhedral-euhedral minerals were also observed, mostly in lower layers. These mineral particles clumped together to coalesce and form irregular aggregates (Figure 8G). Occasionally, some cells were observed encrusted within minerals.

Ca, S, and Si were revealed as major elements (other than light elements such as C, O, and N) within the microbial mat by EDS-elemental mapping (Supplementary Figure S2). Abundant calcium was present as part of the aggregates previously visualized by SEM, suggesting that these are composed of calcium carbonate minerals. Calcite (CaCO_3_) was confirmed by XRD analysis as the major mineral phase all along the microbial mat, without distinction between layers (Supplementary Figure S3). In addition, nanometer-sized sulfur globules were observed inside bacterial cells by EDS mapping, suggesting that sulfur metabolizing bacteria were present, consistently with 16S rRNA gene analyses. Silica was mainly observed as part of the diatoms present in the mat.

### 3.4. Confocal Laser Scanning Microscopy Analyses 

**Figure 9:**
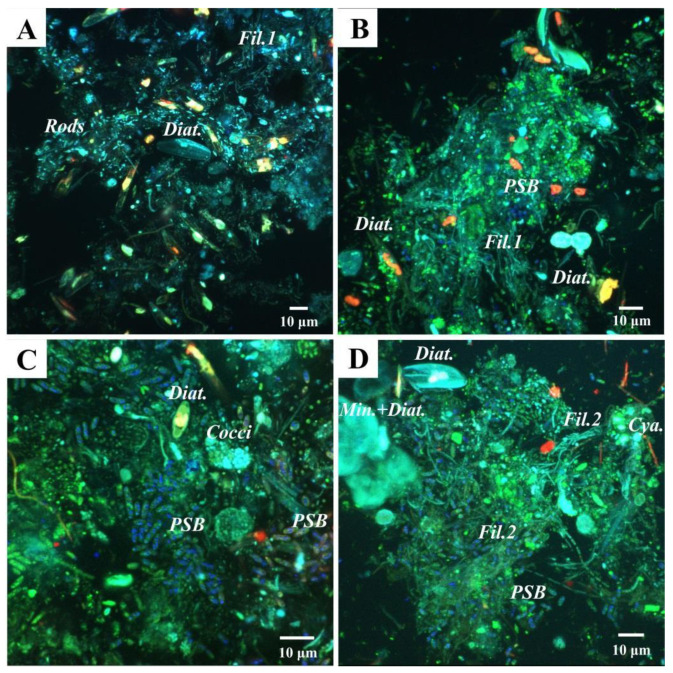
Confocal Laser Scanning Microscopy (CLSM) images of the studied microbial mat layers. (**A**,**B**) Top layer of the mat, where (**A**) shows a fixed sample without staining and (**B**) shows a sample stained with Syto^®^9 (DNA staining). Diatoms are visible in light blue (Diat.), and also rod-shaped bacteria (Rods) and filamentous bacteria (Fil.1). Purple Sulfur Bacteria (PSB) are detected due to laser reflection of the sulfur globules inside the cells in dark blue; (**C**,**D**) CLSM images that correspond to Layers 2 and 3, both stained with Syto^®^9; (**C**) Abundant PSB are visualized, as well as dense colonies of autoflourescent cocci (Cocci), and some diatoms (Diat.); (**D**) Filamentous bacteria, possibly Chloroflexi (Fil.2), diatoms (Diat.), and diatoms frustules associated with globules of minerals (Min. + Diat.) detected in light blue, PSB in dark blue, and a few Cyanobacteria (Cya.) in red, due to chlorophyll a fluorescence.

Diatoms were detected by CLSM as well. Photosynthetic pigment (chlorophyll *a*) was detected in red and the frustules in blue (Figure 9A–D). Occasionally, diatom frustules were found immersed within mineral globules (Figure 9D). In addition to diatoms, another major primary producer group in this mat seems to be the Purple Sulfur Bacteria (PSB), based on the detection of abundant rod-shaped bacteria containing sulfur globules by CLSM (observed in dark blue due to laser reflection; Figure 9B–D). Few Cyanobacteria were visible in red, due to chlorophyll *a* fluorescence (Figure 9D). In addition, a plethora of microorganisms in close relation with the mineral grains were revealed in green after labeling with Syto^®^9 staining. For instance, in Layer 1, fluorescent rod-shaped bacteria and filamentous bacteria were distinguished (Figure 9A,B). In Layers 2 and 3, filamentous bacteria resembling Chloroflexi and coccoid cells forming dense colonies were also found.

## 4. Discussion

### 4.1. Microbial Diversity of the Laguna Negra Microbial Mat and Other Andean Wetlands 

Here, the stratified microbial mat associated with oncoids in the Laguna Negra system was studied in detail, providing new insights about its microbial diversity, functional aspects inferred by diversity and pigments, and mineralogical and textural description of each layer. This study allows a better characterization of the prokaryotic microbial diversity changes at different depths, and thus, significantly improves our understanding of microbial community spatial arrangement in this extreme environment. Bacteroidetes, Deinococcus-Thermus, Verrucomicrobia, Halanaerobiaeota, Proteobacteria, Chloroflexi, and Planctomycetes were found as the most abundant groups. This diversity data supported the previous findings about the stratified mat studied as a bulk by Gomez et al. [39], except for groups under recent revision, such as Firmicutes, OD1, and Halanaerobiaeota. 

There are Andean wetlands microbial communities dominated by Archaea, such as those in Diamante Lake [27], evaporates domes in Tebenquiche [24], top of the non-lithifying microbial mat in La Brava [22], and Peruvian Maras Salterns [66]. Here, it was shown that the LN stratified mat is a bacterial dominated community, although Archaea reaches 5% abundance in bottom layers, suggesting an appreciable contribution by Archaea to the metabolic activities deep in the mat. In terms of bacterial diversity at the phylum level, taxa found here are consistent with previous comparable studies in the Altiplano-Puna region. In this sense, in other Andean wetlands, such as Tebenquiche, La Brava [21,24], Socompa [20,25], Diamante [27], Cejar, Llamara, Jachucoposa, Pujsa [23], Laguna Santa Rosa and Laguna Verde [67], and Salar de Huasco ponds [68], some of the most abundant phyla usually found were Spirochaetes, Deinococcus-Thermus, Planctomycetes, Firmicutes, Verrucomicrobia, Parcubacteria, Chloroflexi, and Actinobacteria, but more noticeable and consistently, Proteobacteria and Bacteroidetes. Overall, these similarities might be related to the similar extreme environmental conditions prevailing all over the high-altitude Andean water systems.

Interestingly, Cyanobacteria have been reported in very low to low abundance in most of these microbial systems at high-altitude [21,23,24,25,27,69], except for singular cases such as in Laguna de la Piedra [70], a red pond in Salar de Huasco [68], or a peculiar black pustular microbial mat from the LN, where Cyanobacteria were abundant [39,71,72]. In the surface layer of the microbial mat studied here, only 3% of the reads belonged to Cyanobacteria. It is worth noticing that there was a considerable Chl *a* content along the mat, with the same depth profile as fucoxanthin (diatom specific accessory pigment), suggesting that the oxygenic photosynthetic guild was dominated by diatoms, a conspicuous group present in the LN microbial mats (visible by SEM, CLSM, revealed by diatoxanthin, diadinoxanthin and fucoxanthin content), and reported as the main algal group in South American high-altitude aquatic systems [73,74].

### 4.2. Microbial Taxa and Pigments Related with High UV Radiation in the Top Layer 

Abundant Deinococcus-Thermus (all Trueperaceae family, and two abundant ASVs assigned to genus *Truepera* sp.), deinoxanthin, carotenoid and potentially MAAs content found at the top of the microbial mat are all related with a high UV-stress prevailing at the surface of the mat and provide its characteristic pink-orange color. The Deinococcus-Thermus phylum contains species belonging to the *Deinococcus* and *Truepera* genera that have been studied as models for resistance to ionizing radiation, including UV radiation. This resistance is partly provided by the presence of deinoxanthin and other compounds [56,75,76,77,78,79]. The Deinococcus-Thermus phylum has also been found in surface layers of other high-altitude microbialite systems where UV light influx is high in the Altiplano-Puna region [28] (such as La Brava, Socompa, and Tebenquiche), as well as in the Obsidian Pool siliceous stromatolites in the Yellowstone Plateau, placed at 2400 m.a.s.l. [80]. In contrast, this phylum is found in minor abundance in low-altitude microbialite systems, for example in Guerrero Negro [81], Shark Bay [82], and Highborne Cay [83], where UV radiation is lower. Therefore, our results support the idea that representatives of this phylum populating the top layer of the microbial mat protect the rest of the community from UV radiation in high-altitude systems, as suggested by Farías et al. [20] and Fernandez et al. [24].

Among the pigments extracted by methanol from the top layer exclusively, pigments showing broad and intense absorbance peak between 310–360 nm were detected. This corresponds to the range of absorbance by MAAs. These are photoprotectors acting as a passive screen dissipating the absorbed UV energy as heat. They are also known for their antioxidant, osmotic regulation, and nitrogen reservoir capabilities [84,85,86,87]. The MAAs are present in a wide range of organisms exposed to high light intensity in marine, freshwater, and terrestrial environments: cyanobacteria, microalgae, fungi, invertebrates, vertebrates [87], and diatoms [88]. Therefore, it is hypothesized that the MAAs present in the LN microbial mat have UV-protective characteristics, and might be related with diatoms, given the conspicuity of these microorganisms. As a final point about Layer 1, it is worth mentioning that indexes such as CHAO1, observed species, and Shannon had the lowest values, and the layer had the highest proportion of unique ASVs. This is likely the result of the selective pressure exerted by a high environmental stress, i.e., UV radiation that selects microorganisms specifically tolerant to this condition. A similar depth distribution of richness was observed in Socompa’s stromatolites [25], and in the La Brava Lake non-lithifying mat [22].

### 4.3. The Laguna Negra Microbial Mat Community Harbors Halophilic Members of Chloroflexi and Halanaerobiaeota 

Halophilic anoxygenic phototrophs in the microbial mat were revealed by diversity and pigment analyses as well. Layer 2 was markedly rich in Bchl *a*, *c*, and *d*, and Chloroflexi (green/filamentous non-sulfur bacteria) and, in lesser proportion, Chromatiaceae (Gammaproteobacteria, which include purple sulfur bacteria) reached their highest abundance in Layer 2 as well. Here, Bchl *c* and *d* could be related to Chloroflexi, since Chlorobi was undetected. The genus *Candidatus* Chlorothrix (unclassified species) represented almost all Chloroflexi found in the mat. The only member studied from this genus is *Candidatus* Chlorothrix halophila, a photoautotrophic bacterium obtained from an enrichment culture from a hypersaline microbial mat community in Guerrero Negro [89,90]. Given that this strain contains Bchl *a* and *c* only, the presence of Bchl *d* in the stratified mat suggests that the Chlorothrix strain inhabiting the LN mat might be different, although more studies are needed to elucidate this issue. On the other hand, Chromatiaceae comprises representatives with photo/chemolithoautotrophic capabilities, salt-requirements, and Bchl *a* content [91]. Moreover, Chromatiaceae is known for the intracellular storage of sulfur globules [92,93]. This is consistent with the detection of abundant elemental sulfur granules, mainly in Layer 2, by SEM-EDS mappings, CLSM, and methanol extraction. Alternatively, other non-phototrophic sulfur-oxidizing bacteria (SOBs) are known for elemental sulfur storage. In this work, Epsilonbacteraeota, mainly Helicobacteraceae, a family that includes environmental SOBs members, were found especially in Layer 2 [94].

Deeper in the mat, halophilic anaerobes were detected. Deltaproteobacteria were the most prevalent Proteobacteria populating bottom layers, with the Oligoflexales order (candidate family 0319-6G20) as the main group among Deltaproteobacteria in layer 3, and Desulfobacteraceae extended in Layers 3 and 4. Little is known about this candidate group of Oligoflexales, and the order systematics is under revision [95,96]. As the diversity was evaluated deeper in the mat, Halanaerobiaeota was found gradually increasing in abundance and becoming the most abundant phylum in Layer 4, with three abundant ASVs assigned to *Halanaerobium* sp. In correspondence with the environmental conditions present in the LN and the layer in which they were found, members of this genus are known for being anaerobic and extremely halophilic [97], some of them being fermenters [98,99], and others sulfur reducers [100,101,102]. Desulfobacteraceae is well known for encompassing sulfur reducing members, but since this family did not prevail in bottom layers as much as Halanaerobiaeota, it is hypothesized that the sulfur reduction guild is composed mainly of Halanaerobiaeota in the LN microbial mat.

### 4.4. Aggregates as Main Structural Component with Geomicrobiological Implications

Finally, subspherical aggregates composed of diatoms, calcite, EPS, and prokaryotic cells were observed without significant changes all along the layers in the stratified microbial mat. Distinctive stratification in hypersaline phototrophic microbial mats in terms of pH, O_2_, H_2_S, light, as well as microbial diversity is well known [3,103,104], and numerical modeling studies aiming at understanding biogeochemical cycling within these mats are based on these features [105,106,107]. In spite of these, clustering or aggregation of microbial consortia within layered microbial mats may produce a more complex pattern in the distribution of biogeochemical zones. This sort of micro-niche has already been studied in ecosystems where some sulfate-reducing bacteria create anoxic microenvironments by forming clusters [108,109,110,111], thus tolerating and populating the predominately oxic zone within the mat or sediment. As observed here, in parallel to the vertical distribution of the prokaryotic microbial diversity, there is also a specific distribution and arrangement of microorganisms at a smaller scale in these aggregates (as suggested by confocal microscopy studies, refs. [39,71], this work), locally changing the chemical balance within the mat. This has particular relevance for geomicrobiological studies of microbialites formation and biosignatures, since carbonate precipitation may take place within these aggregates, influencing the textures and geochemical signatures of the formed carbonate phases. This aspect deserves further studies, for example by using nano-scale chemical mapping techniques (such as nano-SIMS) that could be useful to have a deeper understanding of the (trace) chemical heterogeneity due to the presence of these aggregates.

## 5. Conclusions

Overall, this is the first diversity survey that approaches the widespread microbial mat of the Laguna Negra by layers, studying each stratum individually. It was shown that the first layer has a distinguished UV-tolerance feature, characterized by the presence of Deinococcus-Thermus, deinoxanthin, carotenoid, and potentially MAAs, which might reflect a shielding strategy to cope with high UV radiation. In addition, a better understanding of the phototrophic, sulfur related, and extremely halophilic communities of the microbial mat was provided, finding Bchl *a*, *c*, and *d* and Chloroflexi in the second layer, and a significant proportion of Halanaerobiaeota in the bottom layers. Given all the unclassified taxa found in this microbial mat, we consider that the Laguna Negra represents a unique and promising extreme environment with great potential for the discovery of new high UV tolerants and halophiles. Finally, subspherical aggregates composed of diatoms, calcite, EPS, and other microorganisms were found all along the layers as a main structural component, which might represent hot spots for carbonate precipitation in this system; however, further studies are needed to reveal mineral precipitation at this smaller scale, and their distinctive biosignatures in microbialites.

## Figures and Tables

**Table 1:** Pigment content of the layers of the microbial mat detected by high-performance liquid chromatography (HPLC) *.

Retention Time (min)	Pigment	Layer 1	Layer 2	Layer 3	Layer 4	Related Organisms According to Bibliography
3.08	Unknown	-	-	-	X	
5.067	Fucoxanthin	XX	-	XXX	XXX	Diatoms, brown algae [58]
6.883	Diadinoxanthin	XX	X	-	-	Diatoms, phaeophytes, dinophytes, haptophytes [58]
7.547	Bacteriochlorophyll *d*	-	XXX	XX	-	Chloroflexi, Chlorobi [59]
7.553	Deinoxanthin	XX	-	-	-	*Deinococcus* spp. [60]
7.85	Carotenoid	X	-	-	-	Algae, diatoms, bacteria, plants, fungi [61]
8.187	Diatoxanthin	XXX	X	XXX	XXX	Diatoms, phaeophytes, dinophytes, haptophytes [58]
8.417	Zeaxanthin	X	-	XX	-	Algae, diatoms, bacteria [58], plants [62]
9.151	Bacteriochlorophyll *c*	-	XXX	XXX	X	Chloroflexi, Chlorobi, Acidobacteria [59]
9.2	Cantaxanthin	X	-	-	-	Bacteria (Acidobacteria) [59], algae, fungi, plants [63]
9.504	Bacteriochlorophyll *d* derivative	-	XX	X	-	
9.784	Bacteriochlorophyll *a*	-	XXXX	X	X	Alpha, Beta, and Gamma-proteobacteria, Chloroflexi, Gemmatimonadota [59], Chlorobi [64]
10.283	Bacteriochlorophyll *a* derivative	-	-	XX	XXX	
10.787	(2S,29S)-oscillol 2,29-difucoside (match 96%)	-	XX	-	-	Cyanobacteria [57]
11.633	Chlorophyll *a*	XXX	-	XX	XXX	Cyanobacteria, diatoms, plants [58]
13.882	Pheophytin	-	-	-	XXX	Bacteria, algae, plants [65]
14.084	Betacarotene	-	X	-	-	Bacteria (Chloroflexi, Acidobacteria) [59], algae, diatoms, plants [58]

* X: OD < 0.01; XX: 0.01 < OD < 0.02; XXX: OD > 0.02; XXXX: OD > 0.2.

## Data Availability

Metadata of sampled layers and 16S rRNA gene reads were submitted to NCBI under the Bioproject PRJNA564857.

## Supplementary Materials

The following supporting information can be downloaded at: https://www.mdpi.com/article/10.3390/biology11060831/s1. Supplementary Figure S1: Rarefaction curve of Observed ASVs; Supplementary Figure S2: EDS maps; Supplementary Figure S3: XRD spectra; Supplementary Tables S1–S6: Descriptive statistics of Alpha Indexes; Supplementary Table S7: Most abundant ASVs [41,51,52,97,112,113,114,115,116,117,118,119,120,121,122,123,124].
