# Supplementary material for: Community Vertical Composition of the Laguna Negra Hypersaline Microbial Mat, Puna Region (Argentinean Andes)
Source: Biology (Basel). 2022 May 28;11(6):831. doi: 10.3390/biology11060831 (PMC9220024; doi:10.3390/biology11060831)
Supplement: Supplementary file 1 [file biology-11-00831-s001.zip › biology-1733266-SI.pdf]

SUPPLEMENTARY MATERIALS

Community Vertical Composition of the Laguna Negra Hypersaline Microbial Mat, Puna Region (Argentinean Andes)

Flavia Jaquelina Boidi [1,*], Estela Cecilia Mlewski [2], Guillermo César Fernández [3], María Regina Flores [4], Emmanuelle Gérard [5], María Eugenia Farías [6] and Fernando Javier Gomez [3,*]

[1] Laboratorio de Biología Molecular, Hospital SAMCo Rafaela "Dr. Jaime Ferré", 737 Lisandro de la Torre, Rafaela 2300, Argentina; flajboidi@gmail.com
[2] Instituto Multidisciplinario de Biología Vegetal (IMBIV), Facultad de Ciencias Exactas, Físicas y Naturales, Universidad Nacional de Córdoba, CONICET, 1666 Vélez Sarsfield Av., Córdoba 5000, Argentina; ecmlewski@imbiv.unc.edu.ar
[3] Centro de Investigaciones en Ciencias de la Tierra (CICTERRA), Facultad de Ciencias Exactas, Físicas y Naturales, Universidad Nacional de Córdoba, CONICET, 1699 Vélez Sarsfield Av., Córdoba 5000, Argentina; guillefernandez.unc@gmail.com (G.C.F.); fjgomez@unc.edu.ar (F.J.G.)
[4] Department of Research and Development, PROMICOL B.V., 12 De Asselen Kuil, Geleen 6161, Netherlands; acm_regy@hotmail.com
[5] Université de Paris Cité, Institut de physique du globe de Paris, CNRS, F-75005 Paris, France; emgerard@ipgp.fr
[6] Planta Piloto de Procesos Industriales y Microbiológicos (PROIMI), Centro Científico Tecnológico – CONICET, Belgrano Av. & Pasaje Caseros, San Miguel de Tucumán 4000, Argentina; mefarias2009@gmail.com
* Correspondence: flajboidi@gmail.com (F.J.B.); fjgomez@unc.edu.ar (F.J.G)

SUPPLEMENTARY FIGURES

Supplementary Figure S1. Observed ASVs rarefaction curve. Rarefaction curves were obtained by multiple repeated sub-sampling (1000 times). For each sampling depth the 1000 calculated value are plotted as circles and the data dispersion is showed in boxplots.

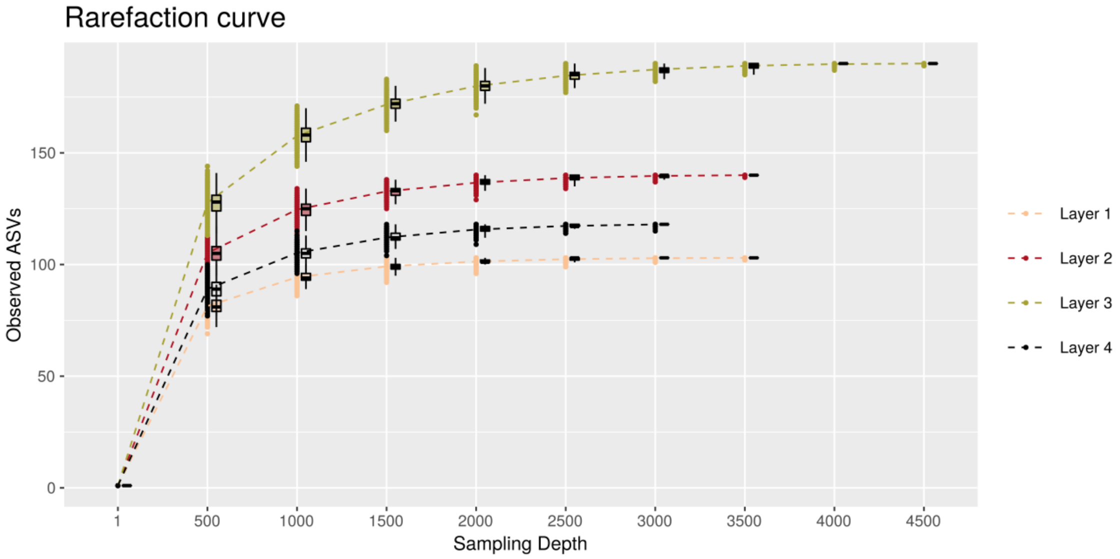

Supplementary Figure S2. EDS maps where major elements distribution is visualized. Note Ca as part of aggregates (*Aggr*.), S forming globules (*SG*.) and Si as part of diatoms (*Diat*.).

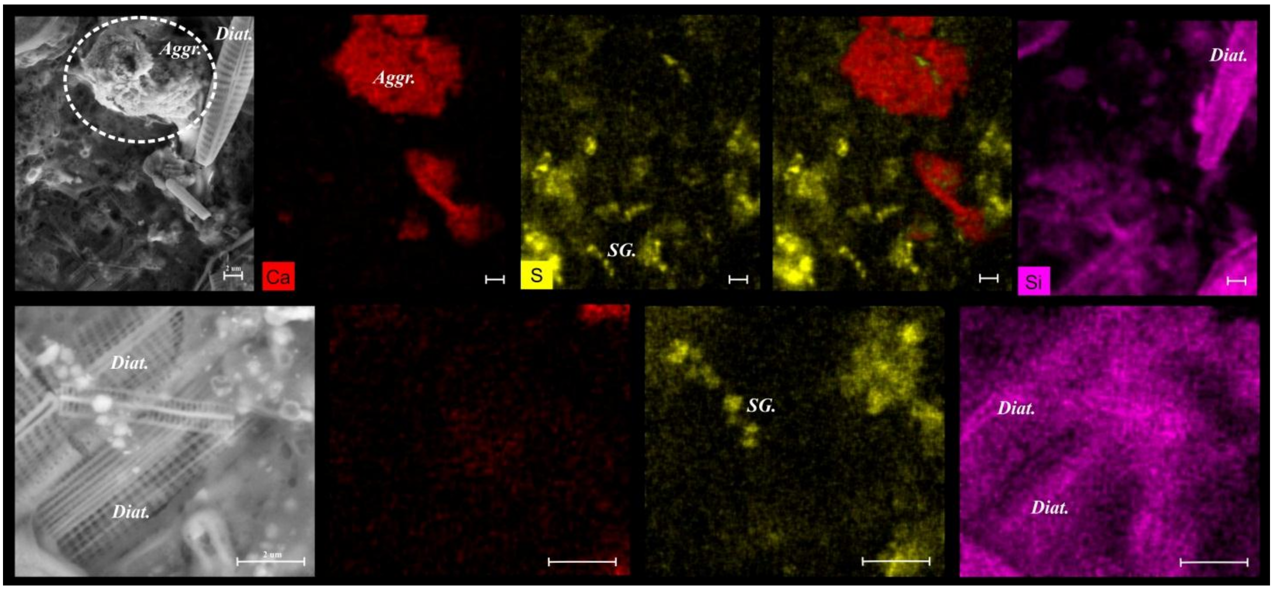

Supplementary Figure S3. XRD spectra showing calcite pattern with red lines in all layers.

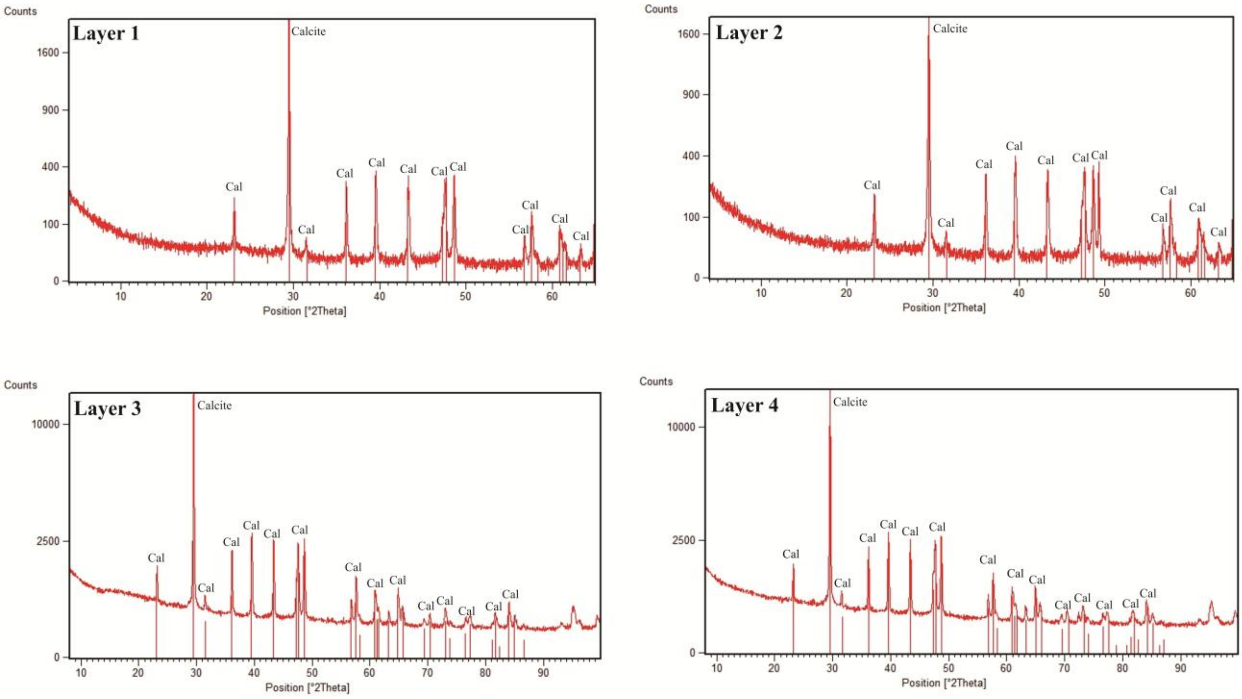

SUPPLEMENTARY TABLES

Supplementary Table S1. Descriptive statistics of Observed ASVs analysis. Values were obtained using a sub-sampling quota of 3000 reads per sample. The sub-sampling routine was performed for 1000 iterations.

Observed ASVs					
**Layer**	**N**	**Mean**	**Standard Deviation**	**Standard Error**	**Confident Intervals (95%)**
**1**	1000	102.830	0.4065283	0.012855554	0.02522699
**2**	1000	139.675	0.5794055	0.018322410	0.03595482
**3**	1000	187.319	1.5848504	0.050117370	0.09834739
**4**	1000	117.941	0.2521559	0.007973869	0.01564745

Non-parametric statistical analysis of Observed ASVs

(Kruskal-Wallis test, H_3 = 3862.3082, *p-value* < 0.0001).

Dunn's test: Layer 1 vs Layer 2 (*p-value* < 0.0001), Layer 1 vs Layer 3 (*p-value* < 0.0001), Layer 1 vs Layer 4 (*p-value* < 0.0001), Layer 2 vs Layer 3 (*p-value* < 0.0001), Layer 2 vs Layer 4 (*p-value* < 0.0001), Layer 3 vs Layer 4 (*p-value* < 0.0001).

Supplementary Table S2. Descriptive statistics of Shannon index analysis. Values were obtained using a sub-sampling quota of 3000 reads per sample. The sub-sampling routine was performed for 1000 iterations

Shannon					
**Layer**	**N**	**Mean**	**Standard Deviation**	**Standard Error**	**Confident Intervals**
1	1000	5.451751	0.01325192	0.0004190624	0.0008223434
2	1000	5.887495	0.01467669	0.0004641178	0.0009107576
3	1000	6.488320	0.01911901	0.0006045961	0.0011864240
4	1000	5.452609	0.01176552	0.0003720584	0.0007301056

Non-parametric statistical analysis of Shannon index

(Kruskal-Wallis test, H_3 = 3374.5, *p-value* < 0.0001).

Dunn's test: Layer 1 vs Layer 2 (*p-value* < 0.0001), Layer 1 vs Layer 3 (*p-value* < 0.0001), Layer 1 vs Layer 4 (*p-value* < 0.0001), Layer 2 vs Layer 3 (*p-value* < 0.0001), Layer 2 vs Layer 4 (*p-value* < 0.0001), Layer 3 vs Layer 4 (*p-value* < 0.0001).

Supplementary Table S3. Descriptive statistics of Simpson index analysis. Values were obtained using a sub-sampling quota of 3000 reads per sample. The sub-sampling routine was performed for 1000 iterations.

Simpson					
**Layer**	**N**	**Mean**	**Standard Deviation**	**Standard Error**	**Confident Intervals**
1	1000	0.9591646	0.0005086771	1.608578e-05	3.156580e-05
2	1000	0.9608285	0.0007163063	2.265160e-05	4.445017e-05
3	1000	0.9803524	0.0004251427	1.344419e-05	2.638210e-05
4	1000	0.9434626	0.0007591020	2.400491e-05	4.710583e-05

Non-parametric statistical analysis of Simpson index

(Kruskal-Wallis test, H_3 = 3707.6696, *p-value* < 0.0001).

Dunn's test: Layer 1 vs Layer 2 (*p-value* < 0.0001), Layer 1 vs Layer 3 (*p-value* < 0.0001), Layer 1 vs Layer 4 (*p-value* < 0.0001), Layer 2 vs Layer 3 (*p-value* < 0.0001), Layer 2 vs Layer 4 (*p-value* < 0.0001), Layer 3 vs Layer 4 (*p-value* < 0.0001).

Supplementary Table S4. Descriptive statistics of Dominance index analysis. Values were obtained using a sub-sampling quota of 3000 reads per sample. The sub-sampling routine was performed for 1000 iterations.

**Dominance**					
**Layer**	**N**	**Mean**	**Standard Deviation**	**Standard Error**	**Confident Intervals**
1	1000	0.04083544	0.0005086771	1.608578e-05	3.156580e-05
2	1000	0.03917145	0.0007163063	2.265160e-05	4.445017e-05
3	1000	0.01964765	0.0004251427	1.344419e-05	2.638210e-05
4	1000	0.05653737	0.0007591020	2.400491e-05	4.710583e-05

Non-parametric statistical analysis of Dominance index

(Kruskal-Wallis test, H_3 = 3707.7, *p-value* < 0.0001).

Dunn's test: Layer 1 vs Layer 2 (*p-value* < 0.0001), Layer 1 vs Layer 3 (*p-value* < 0.0001), Layer 1 vs Layer 4 (*p-value* < 0.0001), Layer 2 vs Layer 3 (*p-value* < 0.0001), Layer 2 vs Layer 4 (*p-value* < 0.0001), Layer 3 vs Layer 4 (*p-value* < 0.0001).

Supplementary Table S5. Descriptive statistics of Pielou_e (J) index analysis. Values were obtained using a sub-sampling quota of 58000 reads per sample. The sub-sampling routine was performed for 1000 iterations.

**Pielou_e *(J)***					
**Layer**	**N**	**Mean**	**Standard Deviation**	**Standard Error**	**Confident Intervals**
1	1000	0.8156294	0.002054605	6.497231e-05	0.0001274979
2	1000	0.8262090	0.002079192	6.574983e-05	0.0001290236
3	1000	0.8594608	0.002544751	8.047209e-05	0.0001579137
4	1000	0.7923095	0.001698435	5.370922e-05	0.0001053958

Non-parametric statistical analysis of Pielou_e (*J*) index

(Kruskal-Wallis test, H_3 = 3749, *p-value* < 0.0001).

Dunn's test: Layer 1 vs Layer 2 (*p-value* < 0.0001), Layer 1 vs Layer 3 (*p-value* < 0.0001), Layer 1 vs Layer 4 (*p-value* < 0.0001), Layer 2 vs Layer 3 (*p-value* < 0.0001), Layer 2 vs Layer 4 (*p-value* < 0.0001), Layer 3 vs Layer 4 (*p-value* < 0.0001).

Supplementary Table S6. Descriptive statistics of Chao1 index analysis. Values were obtained using a sub-sampling quota of 3000 reads per sample. The sub-sampling routine was performed for 1000 iterations.

Chao1					
**Layer**	**N**	**Mean**	**Standard Deviation**	**Standard Error**	**Confident Intervals**
**1**	1000	103.0906	0.6621615	0.02093939	0.04109023
**2**	1000	140.2106	0.8781844	0.02777063	0.05449546
**3**	1000	191.0313	2.8453931	0.08997923	0.17656997
**4**	1000	118.0717	0.3468058	0.01096696	0.02152092

Non-parametric statistical analysis of Chao 1 index

(Kruskal-Wallis test, H_3 = 3749.1, *p-value* < 0.0001).

Dunn's test: Layer 1 vs Layer 2 (*p-value* < 0.0001), Layer 1 vs Layer 3 (*p-value* < 0.0001), Layer 1 vs Layer 4 (*p-value* < 0.0001), Layer 2 vs Layer 3 (*p-value* < 0.0001), Layer 2 vs Layer 4 (*p-value* < 0.0001), Layer 3 vs Layer 4 (*p-value* < 0.0001).

Supplementary Table S7. Most abundant ASVs (>0.02 relative abundance) per layer classified at the lowest possible taxonomic level. Known genera are highlighted in bold.

**ASV**	**Layer**	**Relative abundance**	**Silva predicted taxonomy**		**NCBI best BLAST match (Accession Number)**	**Identity**	**Isolation source**	**Reference**
**01**	1	0.00	Bacteria	Phylum Halanaerobiaeota<br>Class Halanaerobiia<br>Order Halanaerobiales<br>Family Halanaerobiaceae<br>Genus ***Halanaerobium***<br>Species Unknown	Uncultured *Halanaerobium* sp.<br>(FM879119.1)	100 %	Hyperhaline lake sediment,<br>Salar de Ascotán, Chile	[53]
	2	0.03						
	3	0.05						
	4	0.19						
**02**	1	0.00	Bacteria	Phylum Thermotogae<br>Class Thermotogae<br>Order Petrotogales<br>Family Petrotogaceae<br>Genus SC103<br>Species Uncultured organism	Uncultured organism clone SBYH_2623<br>(JN455679.1)	98.8 %	Hypersaline microbial mat,<br>Guerrero Negro, Mexico	[41]
	2	0.02						
	3	0.04						
	4	0.03						
**03**	1	0.04	Bacteria	Phylum Proteobacteria<br>Class Alphaproteobacteria<br>Order Rickettsiales<br>Family Unknown<br>Genus Unknown<br>Species Unknown	Uncultured bacterium clone ProA23S_19<br>(GQ915826.1)	95.6 %	Marine water,<br>Gulf of Mexico, USA	[54]
	2	0.00						
	3	0.00						
	4	0.00						
**04**	1	0.01	Bacteria	Phylum Patescibacteria<br>Class Gracilibacteria<br>Order Absconditabacteriales (SR1)<br>Family Unknown<br>Genus Unknown<br>Species Unknown	Uncultured organism clone SBXZ_5580<br>(JN436702.1)	99.2 %	Hypersaline microbial mat,<br>Guerrero Negro, Mexico	[41]
	2	0.04						
	3	0.00						
	4	0.00						
**05**	1	0,03	Bacteria	Phylum Verrucomicrobia<br>Class Verrucomicrobiae<br>Order Opitutales<br>Family Puniceicoccaceae<br>Genus ***Lentimonas***<br>Species Uncultured Verrucomicrobia	Uncultured bacterium clone 59_1854298<br>(LT720392.1)	100 %	Marine water,<br>Rio de Janeiro, Brazil	[55]
	2	0.00						
	3	0.01						
	4	0.00						
**06**	1	0.00	Bacteria	Phylum Proteobacteria<br>Class Deltaproteobacteria<br>Order Oligoflexales<br>Family 0319-6G20<br>Genus Unknown<br>Species Unknown	Uncultured organism clone SBYH_6277<br>(JN459997.1)	99.6 %	Hypersaline microbial mat,<br>Guerrero Negro, Mexico	[41]
	2	0.01						
	3	0.07						
	4	0.03						

Supplementary Table S7. Most abundant ASVs (>0.02 relative abundance) per layer classified at the lowest possible taxonomic level. Known genera are highlighted in bold. (continued)

ASV	Layer	Relative abundance	Silva predicted taxonomy		NCBI best BLAST match (Accession Number)	Identity	Isolation source	Reference
**07**	1	0.08	Bacteria	Phylum Deinococcus-Thermus<br>Class Deinococci<br>Order Deinococcales<br>Family Trueperaceae<br>Genus ***Truepera***<br>Species Uncultured organism	Uncultured bacterium clone PET4-24 (MG949154.1)	99.6 %	Hypersaline microbial mat, Sečovlje Salina, Slovenia	[56]
	2	0.00						
	3	0.00						
	4	0.00						
**08**	1	0.05	Bacteria	Phylum Patescibacteria<br>Class Gracilibacteria<br>Order Absconditabacteriales (SR1)<br>Family Uncultured bacterium<br>Genus Uncultured bacterium<br>Species Uncultured bacterium	Uncultured candidate division SR1 bacterium clone Elkhorn-918R-1A (JF917294.1)	98.8 %	Elkhorn slough, California, USA	[57]
	2	0.03						
	3	0.00						
	4	0.00						
**09**	1	0.00	Bacteria	Phylum Planctomycetes<br>Class SGST604<br>Order Uncultured organism<br>Family Uncultured organism<br>Genus Uncultured organism<br>Species Uncultured organism	Uncultured organism clone SBZC_540 (JN508204.1)	98 %	Hypersaline microbial mat, Guerrero Negro, Mexico	[41]
	2	0.02						
	3	0.00						
	4	0.03						
**10**	1	0.00	Bacteria	Phylum Spirochaetes<br>Class Leptospirae<br>Order Leptospirales<br>Family Leptospiraceae<br>Genus Uncultured<br>Species Uncultured organism	Uncultured organism clone SBYO_2277 (JN466486.1)	99.6 %	Hypersaline microbial mat, Guerrero Negro, Mexico	[41]
	2	0.01						
	3	0.03						
	4	0.02						
**11**	1	0.00	Bacteria	Phylum Thermotogae<br>Class Thermotogae<br>Order Petrotogales<br>Family Petrotogaceae<br>Genus SC103<br>Species Uncultured organism	Uncultured organism clone SBXZ_2085 (JN432239.1)	98.8 %	Hypersaline microbial mat, Guerrero Negro, Mexico	[41]
	2	0.00						
	3	0.03						
	4	0.00						
**12**	1	0.00	Bacteria	Phylum Patescibacteria<br>Class Gracilibacteria<br>Order Absconditabacteriales (SR1)<br>Family Unknown<br>Genus Unknown<br>Species Unknown	Uncultured eubacterium WCHA2-26 (AF050609.1)	93.2 %	Contaminated aquifer, Michigan, USA	[58]
	2	0.00						
	3	0.02						
	4	0.00						
**13**	1	0.00	Bacteria	Phylum Planctomycetes<br>Class Unknown<br>Order Unknown<br>Family Unknown<br>Genus Unknown<br>Species Unknown	Uncultured bacterium clone Kasin-B1-B10 (HE604774.1)	92 %	Hypersaline sediment, Lake Kasin, Russia	[59]
	2	0.00						
	3	0.00						
	4	0.02						
**14**	1	0.04	Bacteria	Phylum Bacteroidetes<br>Class Bacteroidia<br>Order Unknown<br>Family Unknown<br>Genus Unknown<br>Species Unknown	Uncultured bacterium clone wat-80 (KC508773.1)	88.1 %	Seawater, Tateyama, Japan	[60]
	2	0.04						
	3	0.00						
	4	0.00						
**15**	1	0.00	Bacteria	Phylum Patescibacteria<br>Class Gracilibacteria<br>Order Candidatus Peregrinibacteria<br>Family Unknown<br>Genus Unknown<br>Species Unknown	Uncultured bacterium clone OM_int_bact086 (KY342779.1)	99.6 %	Hypersaline intertidal microbial mats, Gulf of Oman, Arabian Sea	[61]
	2	0.01						
	3	0.03						
	4	0.02						
**16**	1	0.00	Bacteria	Phylum Halanaerobiaeota<br>Class Halanaerobiia<br>Order Halanaerobiales<br>Family Halanaerobiaceae<br>Genus ***Halanaerobium***<br>Species bacterium YC-ZSS-LKJ30	Uncultured bacterium clone Kasin-B2-F08 (HE604688.1)	99.6 %	Hypersaline sediment, Lake Kasin, Russia	[59]
	2	0.00						
	3	0.03						
	4	0.09						

Supplementary Table S7. Most abundant ASVs (>0.02 relative abundance) per layer classified at the lowest possible taxonomic level. Known genera are highlighted in bold. (continued)

ASV	Layer	Relative abundance	Silva predicted taxonomy		NCBI best BLAST match (Accession Number)	Identity	Isolation source	Reference
**17**	1	0.00	Bacteria	Phylum Epsilonbacteraeota<br>Class Campylobacteria<br>Order Campylobacterales<br>Family Helicobacteraceae<br>Genus Uncultured<br>Species Uncultured organism	Uncultured organism clone SBZP_5796 (JN538963.1)	99.6 %	Hypersaline microbial mat, Guerrero Negro, Mexico	[41]
	2	0.02						
	3	0.00						
	4	0.00						
**18**	1	0.07	Bacteria	Phylum Deinococcus-Thermus<br>Class Deinococci<br>Order Deinococcales<br>Family Trueperaceae<br>Genus ***Truepera***<br>Species Uncultured bacterium	Uncultured bacterium clone Hua-w/2-88 (EF632878.2)	100 %	Hypersaline water, Salar de Huasco, Chile	[62]
	2	0.00						
	3	0.00						
	4	0.00						
**19**	1	0.00	Bacteria	Phylum Atribacteria<br>Class JS1<br>Order Uncultured organism<br>Family Uncultured organism<br>Genus Uncultured organism<br>Species Uncultured organism	Uncultured organism clone SBYZ_1653 (JN496325.1)	99.2 %	Hypersaline microbial mat, Guerrero Negro, Mexico	[41]
	2	0.00						
	3	0.01						
	4	0.04						
**20**	1	0.00	Bacteria	Phylum Halanaerobiaeota<br>Class Halanaerobiia<br>Order Halanaerobiales<br>Family Halanaerobiaceae<br>Genus ***Halanaerobium***<br>Species Unknown	*Halanaerobium praevalens* strain GSL (NR_074859.1)	100 %	Great Salt Lake sediment, Utah, USA	[63]
	2	0.02						
	3	0.00						
	4	0.03						
**21**	1	0.00	Bacteria	Phylum Chloroflexi<br>Class Chloroflexia<br>Order Chloroflexales<br>Family Chloroflexaceae<br>Genus Candidatus ***Chlorothrix***<br>Species Unknown	Uncultured bacterium clone 1109S3_C07_115 (AB785865.1)	98.4 %	Coastal microbial mats, Schiermonnikoog, Netherlands	[64]
	2	0.15						
	3	0.02						
	4	0.00						
**22**	1	0.12	Bacteria	Phylum Bacteroidetes<br>Class Bacteroidia<br>Order Chitinophagales<br>Family Uncultured<br>Genus Uncultured organism<br>Species Uncultured organism	Uncultured organism clone SBXZ_4804 (JN435991.1)	97.6 %	Hypersaline microbial mat, Guerrero Negro, Mexico	[41]
	2	0.00						
	3	0.00						
	4	0.00						
**23**	1	0.00	Bacteria	Phylum Unknown<br>Class Unknown<br>Order Unknown<br>Family Unknown<br>Genus Unknown<br>Species Unknown	Uncultured bacterium clone 1109S3_E03_088 (AB784120.1)	95.7 %	Coastal microbial mats, Schiermonnikoog, Netherlands	[64]
	2	0.06						
	3	0.01						
	4	0.00						
**24**	1	0.03	Bacteria	Phylum Cyanobacteria<br>Class Sericytochromatia<br>Order Uncultured bacterium<br>Family Uncultured bacterium<br>Genus Uncultured bacterium<br>Species Uncultured bacterium	Uncultured bacterium clone cafs740 (MF439907.1)	90.8 %	Floodplain lake water, Lago Grande do Curuai, Brazil	[65]
	2	0.00						
	3	0.00						
	4	0.00						
**25**	1	0.06	Bacteria	Phylum Verrucomicrobia<br>Class Verrucomicrobiae<br>Order Methylacidiphilales<br>Family Methylacidiphilaceae<br>Genus Uncultured<br>Species Uncultured	Uncultured organism clone SBYO_1345 (JN465675.1)	98.8 %	Hypersaline microbial mat, Guerrero Negro, Mexico	[41]
	2	0.05						
	3	0.01						
	4	0.00						
**26**	1	0.06	Bacteria	Phylum Bacteroidetes<br>Class Bacteroidia<br>Order Cytophagales<br>Family Flammeovirgaceae<br>Genus ***Flexithrix***<br>Species Uncultured organism	Uncultured bacterium clone 108280New.CleanUp.RefOTU12935 (MF995916.1)	96 %	Mediterranean thalassohaline lake	[66]
	2	0.00						
	3	0.00						
	4	0.00						

Supplementary Table S7. Most abundant ASVs (>0.02 relative abundance) per layer classified at the lowest possible taxonomic level. Known genera are highlighted in bold. (continued)

ASV	Layer	Relative abundance	Silva predicted taxonomy		NCBI best BLAST match (Accession Number)	Identity	Isolation source	Reference
**27**	1	0.00	Bacteria	Phylum Proteobacteria<br>Class Deltaproteobacteria<br>Order Oligoflexales<br>Family 0319-6G20<br>Genus Uncultured organism<br>Species Uncultured organism	Uncultured organism clone SBZP_879 (JN535274.1)	99.6 %	Hypersaline microbial mat, Guerrero Negro, Mexico	[41]
	2	0.01						
	3	0.03						
	4	0.01						
**28**	1	0.00	Bacteria	Phylum Thermotogae<br>Class Thermotogae<br>Order Petrotogales<br>Family Petrotogaceae<br>Genus SC103<br>Species Uncultured organism	Uncultured organism clone SBXZ_2085 (JN432239.1)	99.2 %	Hypersaline microbial mat, Guerrero Negro, Mexico	[41]
	2	0.00						
	3	0.00						
	4	0.02						
